# *N*-(4-Bromo­phen­yl)-3,5-dinitro­benzamide

**DOI:** 10.1107/S1600536811051464

**Published:** 2011-12-03

**Authors:** Sohail Saeed, Naghmana Rashid, Rizwan Hussain, Wing-Tak Wong

**Affiliations:** a Chemistry Department, Research Complex, Allama Iqbal Open University, Islamabad 44000, Pakistan; b National Engineering & Scientific Commission, PO Box 2801, Islamabad, Pakistan; c Department of Chemistry, The University of Hong Kong, Pokfulam Road, Pokfulam, Hong Kong SAR, People’s Republic of China

## Abstract

The title mol­ecule, C_13_H_8_BrN_3_O_5_, is slightly twisted, with the dihedral angle between the two benzene rings being 5.9 (1)°. In the crystal, N—H⋯O hydrogen bonds link the mol­ecules into one-dimensional chains running along [101]. Further stabilization of the crystal structure is provided by π–π inter­actions [shortest centroid–centroid distance = 3.6467 (17) Å].

## Related literature

For background to the biological activity of *N*-substituted benzamides, their use in synthesis and for related structures, see: Saeed *et al.* (2011*a* ▶,*b* ▶).
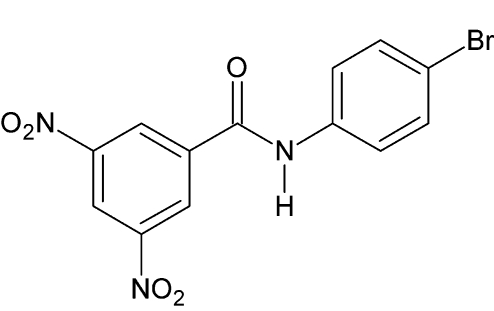


## Experimental

### 

#### Crystal data

C_13_H_8_BrN_3_O_5_
*M* *_r_* = 366.13
Monoclinic, 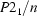

*a* = 7.1273 (2) Å
*b* = 26.6676 (7) Å
*c* = 7.5428 (2) Å
β = 101.652 (2)°
*V* = 1404.10 (7) Å^3^
*Z* = 4
Mo *K*α radiation
μ = 2.96 mm^−1^
*T* = 296 K
0.56 × 0.34 × 0.30 mm

#### Data collection

Bruker APEXII CCD diffractometer
Absorption correction: multi-scan (*SADABS*; Sheldrick, 1996 ▶) *T*_min_ = 0.288, *T*_max_ = 0.471
18250 measured reflections
2476 independent reflections
2007 reflections with *I* > 2σ(*I*)
*R*_int_ = 0.060

#### Refinement

*R*[*F* ^2^ > 2σ(*F* ^2^)] = 0.035
*wR*(*F* ^2^) = 0.108
*S* = 1.10
2476 reflections
204 parameters
H atoms treated by a mixture of independent and constrained refinement
Δρ_max_ = 0.25 e Å^−3^
Δρ_min_ = −0.46 e Å^−3^

### 

Data collection: *APEX2* (Bruker, 2007 ▶); cell refinement: *SAINT* (Bruker, 2007 ▶); data reduction: *SAINT*; program(s) used to solve structure: *SHELXS97* (Sheldrick, 2008 ▶); program(s) used to refine structure: *SHELXL97* (Sheldrick, 2008 ▶); molecular graphics: *Mercury* (Macrae *et al.*, 2008 ▶); software used to prepare material for publication: *SHELXL97*.

## Supplementary Material

Crystal structure: contains datablock(s) I, global. DOI: 10.1107/S1600536811051464/tk5029sup1.cif

Structure factors: contains datablock(s) I. DOI: 10.1107/S1600536811051464/tk5029Isup2.hkl

Supplementary material file. DOI: 10.1107/S1600536811051464/tk5029Isup3.cml

Additional supplementary materials:  crystallographic information; 3D view; checkCIF report

## Figures and Tables

**Table 1:** Hydrogen-bond geometry (Å, °)

*D*—H⋯*A*	*D*—H	H⋯*A*	*D*⋯*A*	*D*—H⋯*A*
N1—H1*N*⋯O1^i^	0.93 (3)	1.93 (3)	2.818 (3)	159 (3)

Symmetry code: (i) 
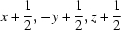
.

**Fig. 1.**
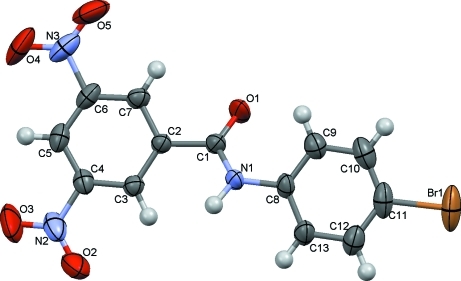
The title molecule showing at the 50% probability displacement ellipsoids and the atom numbering scheme.

**Fig. 2.**
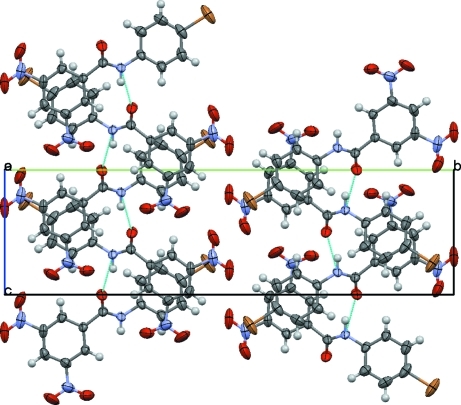
The packing diagram projected down the a axis of the compound showing 50% probability displacement ellipsoids. The cyan dotted lines indicate N—H···O hydrogen bonding interactions.

## supplementary crystallographic information

### Comment

In connection with on-going studies into *N*-substituted benzamides (Saeed *et al.*, 2011*a*,*b*), we recently determined the crystal structure of 3,5-dinitro-*N*-(1,3-thiazol-2-yl)-benzamide monohydrate (Saeed *et al.*, 2011*a*). In this paper we present the crystal structure of the title compound (I), Fig. 1.

Intermolecular N1—H1N···O1 hydrogen bonds link the molecules into 1-D chains running along [101], Table 1 and Fig. 2. The dihedral angle between the two phenyl ring planes is 5.9 (1)°. Both nitro groups are slightly twisted, 3.3 (2)° and 4.6 (2)°, respectively, from the benzene ring plane, C2—C7. There are also weak π–π interactions between neighbouring molecules, Table 2.

### Experimental

To a 250 ml round flask fitted with a condenser was added ethyl 4-bromoaniline (0.1 mol), dichloromethane (15 ml) and triethylamine(0.5 ml) with magnetic stirring. 3,5-Dinitrobenzoyl chloride (0.1 mol) was added gradually. The reaction mixture was stirred at room temperature for 1 h and then refluxed for 2 h. The product precipitated as a colourless powder, which was washed three times with water and dichloromethane. Recrystallization from ethyl acetate produced the crystals of the title compound.

### Refinement

All of the C-bound H atoms are observable in a difference Fourier map but were placed at geometrical positions with C—H = 0.93 Å, and with *U*_iso_(H) = 1.2*U*_eq_(Carrier). The N-bound H-atoms were located from difference Fourier map and refined isotropically.

### Crystal data

**Table:**

C_13_H_8_BrN_3_O_5_	*F*(000) = 728
*M**_r_* = 366.13	*D*_x_ = 1.732 Mg m^−^^3^
Monoclinic, *P*2_1_/*n*	Mo *K*α radiation, λ = 0.71073 Å
Hall symbol: -P 2yn	Cell parameters from 18250 reflections
*a* = 7.1273 (2) Å	θ = 2.9–25.0°
*b* = 26.6676 (7) Å	µ = 2.96 mm^−^^1^
*c* = 7.5428 (2) Å	*T* = 296 K
β = 101.652 (2)°	Block, colourless
*V* = 1404.10 (7) Å^3^	0.56 × 0.34 × 0.30 mm
*Z* = 4	

### Data collection

**Table:**

Bruker APEXII CCD diffractometer	2476 independent reflections
Radiation source: fine-focus sealed tube	2007 reflections with *I* > 2σ(*I*)
graphite	*R*_int_ = 0.060
ω and φ scan	θ_max_ = 25.0°, θ_min_ = 2.9°
Absorption correction: multi-scan (*SADABS*; Sheldrick, 1996)	*h* = −8→8
*T*_min_ = 0.288, *T*_max_ = 0.471	*k* = −31→31
18250 measured reflections	*l* = −8→8

### Refinement

**Table:**

Refinement on *F*^2^	Secondary atom site location: difference Fourier map
Least-squares matrix: full	Hydrogen site location: inferred from neighbouring sites
*R*[*F*^2^ > 2σ(*F*^2^)] = 0.035	H atoms treated by a mixture of independent and constrained refinement
*wR*(*F*^2^) = 0.108	*w* = 1/[σ^2^(*F*_o_^2^) + (0.0472*P*)^2^ + 0.9282*P*] where *P* = (*F*_o_^2^ + 2*F*_c_^2^)/3
*S* = 1.10	(Δ/σ)_max_ < 0.001
2476 reflections	Δρ_max_ = 0.25 e Å^−^^3^
204 parameters	Δρ_min_ = −0.46 e Å^−^^3^
0 restraints	Extinction correction: *SHELXL97* (Sheldrick, 2008), Fc^*^=kFc[1+0.001xFc^2^λ^3^/sin(2θ)]^-1/4^
Primary atom site location: structure-invariant direct methods	Extinction coefficient: 0.0074 (12)

### Special details

**Table:**

Geometry. All e.s.d.'s (except the e.s.d. in the dihedral angle between two l.s. planes) are estimated using the full covariance matrix. The cell e.s.d.'s are taken into account individually in the estimation of e.s.d.'s in distances, angles and torsion angles; correlations between e.s.d.'s in cell parameters are only used when they are defined by crystal symmetry. An approximate (isotropic) treatment of cell e.s.d.'s is used for estimating e.s.d.'s involving l.s. planes.
Refinement. Refinement of *F*^2^ against ALL reflections. The weighted *R*-factor *wR* and goodness of fit *S* are based on *F*^2^, conventional *R*-factors *R* are based on *F*, with *F* set to zero for negative *F*^2^. The threshold expression of *F*^2^ > σ(*F*^2^) is used only for calculating *R*-factors(gt) *etc*. and is not relevant to the choice of reflections for refinement. *R*-factors based on *F*^2^ are statistically about twice as large as those based on *F*, and *R*- factors based on ALL data will be even larger.

### Fractional atomic coordinates and isotropic or equivalent isotropic displacement parameters (Å^2^)

**Table:**

	*x*	*y*	*z*	*U*_iso_*/*U*_eq_	
Br1	0.80856 (6)	0.046874 (16)	0.25109 (7)	0.0884 (2)	
O1	0.6333 (3)	0.28369 (8)	0.5032 (3)	0.0569 (6)	
O2	0.9867 (5)	0.31665 (12)	1.3107 (3)	0.0879 (9)	
O3	0.9761 (6)	0.39563 (13)	1.3387 (4)	0.1162 (14)	
O4	0.8205 (5)	0.50043 (9)	0.8040 (5)	0.0986 (10)	
O5	0.7279 (6)	0.46522 (10)	0.5447 (5)	0.0967 (11)	
N1	0.8777 (3)	0.24105 (8)	0.6787 (3)	0.0395 (6)	
N2	0.9563 (5)	0.35817 (13)	1.2475 (4)	0.0651 (9)	
N3	0.7853 (5)	0.46389 (11)	0.7070 (6)	0.0655 (9)	
C1	0.7638 (4)	0.28096 (10)	0.6368 (4)	0.0393 (6)	
C2	0.8027 (3)	0.32503 (10)	0.7622 (4)	0.0341 (6)	
C3	0.8632 (4)	0.32045 (10)	0.9473 (4)	0.0362 (6)	
H3	0.8834	0.2890	1.0013	0.043*	
C4	0.8932 (4)	0.36369 (11)	1.0508 (4)	0.0430 (7)	
C5	0.8704 (4)	0.41094 (11)	0.9800 (4)	0.0487 (8)	
H5	0.8934	0.4394	1.0524	0.058*	
C6	0.8110 (4)	0.41394 (10)	0.7935 (5)	0.0456 (7)	
C7	0.7736 (4)	0.37237 (10)	0.6849 (4)	0.0405 (6)	
H7	0.7293	0.3759	0.5608	0.049*	
C8	0.8647 (4)	0.19583 (10)	0.5760 (4)	0.0392 (6)	
C9	0.8261 (4)	0.19669 (12)	0.3885 (4)	0.0488 (7)	
H9	0.8107	0.2271	0.3268	0.059*	
C10	0.8108 (5)	0.15204 (15)	0.2943 (5)	0.0580 (9)	
H10	0.7828	0.1522	0.1685	0.070*	
C11	0.8370 (4)	0.10718 (13)	0.3874 (5)	0.0576 (9)	
C12	0.8826 (5)	0.10586 (12)	0.5729 (5)	0.0561 (8)	
H12	0.9045	0.0754	0.6338	0.067*	
C13	0.8955 (4)	0.15065 (11)	0.6683 (4)	0.0481 (8)	
H13	0.9247	0.1504	0.7942	0.058*	
H1N	0.958 (4)	0.2412 (10)	0.792 (4)	0.035 (7)*	

### Atomic displacement parameters (Å^2^)

**Table:**

	*U*^11^	*U*^22^	*U*^33^	*U*^12^	*U*^13^	*U*^23^
Br1	0.0662 (3)	0.0737 (3)	0.1252 (5)	−0.01117 (19)	0.0187 (3)	−0.0669 (3)
O1	0.0609 (13)	0.0435 (11)	0.0499 (13)	0.0067 (10)	−0.0278 (11)	−0.0061 (10)
O2	0.134 (3)	0.084 (2)	0.0409 (14)	−0.0258 (18)	0.0057 (15)	0.0037 (14)
O3	0.205 (4)	0.095 (2)	0.0539 (16)	−0.049 (2)	0.038 (2)	−0.0396 (17)
O4	0.105 (2)	0.0269 (13)	0.161 (3)	−0.0038 (13)	0.020 (2)	−0.0076 (16)
O5	0.133 (3)	0.0518 (16)	0.104 (3)	0.0173 (17)	0.022 (2)	0.0374 (16)
N1	0.0429 (13)	0.0309 (12)	0.0361 (13)	0.0010 (10)	−0.0123 (11)	−0.0037 (10)
N2	0.089 (2)	0.070 (2)	0.0407 (16)	−0.0319 (17)	0.0224 (15)	−0.0123 (15)
N3	0.0619 (19)	0.0326 (15)	0.104 (3)	0.0066 (13)	0.0229 (18)	0.0138 (16)
C1	0.0383 (15)	0.0335 (14)	0.0394 (15)	−0.0015 (11)	−0.0079 (12)	0.0023 (12)
C2	0.0309 (14)	0.0294 (13)	0.0381 (15)	0.0005 (10)	−0.0024 (11)	0.0013 (11)
C3	0.0344 (14)	0.0323 (13)	0.0394 (15)	−0.0027 (11)	0.0016 (11)	0.0020 (11)
C4	0.0448 (16)	0.0465 (16)	0.0389 (16)	−0.0090 (13)	0.0116 (13)	−0.0087 (13)
C5	0.0530 (18)	0.0356 (15)	0.060 (2)	−0.0102 (13)	0.0159 (15)	−0.0130 (14)
C6	0.0410 (16)	0.0261 (14)	0.071 (2)	0.0006 (11)	0.0144 (15)	0.0024 (13)
C7	0.0370 (15)	0.0359 (15)	0.0447 (16)	0.0038 (12)	−0.0012 (12)	0.0058 (13)
C8	0.0331 (14)	0.0350 (14)	0.0440 (16)	−0.0016 (11)	−0.0051 (11)	−0.0108 (12)
C9	0.0471 (17)	0.0522 (18)	0.0446 (18)	−0.0024 (14)	0.0035 (13)	−0.0088 (14)
C10	0.0495 (19)	0.075 (2)	0.0485 (19)	−0.0023 (16)	0.0085 (15)	−0.0248 (18)
C11	0.0373 (17)	0.057 (2)	0.077 (2)	−0.0065 (14)	0.0087 (16)	−0.0346 (18)
C12	0.0495 (19)	0.0402 (16)	0.075 (2)	−0.0007 (14)	0.0032 (16)	−0.0147 (16)
C13	0.0492 (17)	0.0358 (15)	0.0517 (18)	0.0011 (13)	−0.0077 (14)	−0.0076 (13)

### Geometric parameters (Å, °)

**Table:**

Br1—C11	1.898 (3)	C4—C5	1.366 (4)
O1—C1	1.228 (3)	C5—C6	1.387 (5)
O2—N2	1.208 (4)	C5—H5	0.9300
O3—N2	1.205 (4)	C6—C7	1.372 (4)
O4—N3	1.214 (4)	C7—H7	0.9300
O5—N3	1.211 (5)	C8—C9	1.385 (4)
N1—C1	1.337 (4)	C8—C13	1.387 (4)
N1—C8	1.426 (3)	C9—C10	1.380 (5)
N1—H1N	0.93 (3)	C9—H9	0.9300
N2—C4	1.468 (4)	C10—C11	1.381 (5)
N3—C6	1.478 (4)	C10—H10	0.9300
C1—C2	1.499 (4)	C11—C12	1.371 (5)
C2—C3	1.381 (4)	C12—C13	1.388 (4)
C2—C7	1.388 (4)	C12—H12	0.9300
C3—C4	1.385 (4)	C13—H13	0.9300
C3—H3	0.9300		
			
C1—N1—C8	125.1 (2)	C7—C6—C5	122.8 (3)
C1—N1—H1N	116.5 (17)	C7—C6—N3	118.2 (3)
C8—N1—H1N	117.7 (17)	C5—C6—N3	119.0 (3)
O3—N2—O2	123.0 (3)	C6—C7—C2	119.3 (3)
O3—N2—C4	118.1 (3)	C6—C7—H7	120.3
O2—N2—C4	119.0 (3)	C2—C7—H7	120.3
O5—N3—O4	124.9 (3)	C9—C8—C13	120.4 (3)
O5—N3—C6	117.3 (3)	C9—C8—N1	121.2 (3)
O4—N3—C6	117.7 (4)	C13—C8—N1	118.4 (3)
O1—C1—N1	124.4 (3)	C10—C9—C8	119.4 (3)
O1—C1—C2	118.9 (2)	C10—C9—H9	120.3
N1—C1—C2	116.7 (2)	C8—C9—H9	120.3
C3—C2—C7	119.6 (2)	C9—C10—C11	119.8 (3)
C3—C2—C1	123.3 (2)	C9—C10—H10	120.1
C7—C2—C1	117.1 (2)	C11—C10—H10	120.1
C2—C3—C4	118.5 (2)	C12—C11—C10	121.4 (3)
C2—C3—H3	120.7	C12—C11—Br1	120.6 (3)
C4—C3—H3	120.7	C10—C11—Br1	118.0 (3)
C5—C4—C3	123.7 (3)	C11—C12—C13	118.9 (3)
C5—C4—N2	118.4 (3)	C11—C12—H12	120.5
C3—C4—N2	117.8 (3)	C13—C12—H12	120.5
C4—C5—C6	116.0 (3)	C8—C13—C12	120.0 (3)
C4—C5—H5	122.0	C8—C13—H13	120.0
C6—C5—H5	122.0	C12—C13—H13	120.0

### Hydrogen-bond geometry (Å, °)

**Table:**

*D*—H···*A*	*D*—H	H···*A*	*D*···*A*	*D*—H···*A*
N1—H1N···O1^i^	0.93 (3)	1.93 (3)	2.818 (3)	159 (3)

Symmetry codes: (i) *x*+1/2, −*y*+1/2, *z*+1/2.

### Table 2 Table 2. π–π interactions (Å, °) *Cg1* and *Cg2* are centroids of the rings C2-C7 and C8-C13 respectively, *CgI···CgJ* is the distance between ring centroids. The dihedral angle is that between the planes of the rings *I* and *J*. *CgI*_Perp is the perpendicular distance of *CgI* from ring *J*. *CgJ*_Perp is the perpendicular distance of *CgJ* from ring *I*.

**Table:**

*I*	*J*	*CgI···CgJ*	Dihedral angle	*CgI*_Perp	*CgJ*_Perp
1	2^i^	3.7391 (17)	6.24 (14)	3.5945 (11)	-3.6827 (13)
1	2^ii^	3.6467 (17)	6.24 (14)	-3.5254 (12)	3.4038 (13)

symmetry operators: ^i^: -1/2+x, 1/2-y, 1/2+z ^ii^: 1/2+x, 1/2-y, 1/2+z
